# Recovery and Purification of Cutin from Tomato By-Products for Application in Hydrophobic Films

**DOI:** 10.3390/membranes13030261

**Published:** 2023-02-23

**Authors:** Andreia Simões, Isabel M. Coelhoso, Vítor D. Alves, Carla Brazinha

**Affiliations:** 1 LAQV-Requimte, Department of Chemistry, NOVA School of Science and Technology, NOVA University of Lisbon, 2829-516 Caparica, Portugal; 2 LEAF—Linking Landscape, Environment, Agriculture and Food, Associated Laboratory TERRA, Instituto Superior de Agronomia, Universidade de Lisboa, 1349-017 Lisboa, Portugal

**Keywords:** tomato pomace, cutin, fatty acids, chitosan-cutin blend films, food packaging

## Abstract

Tomato pomace is a low-cost, renewable resource that has been studied for the extraction of the biopolyester cutin, which is mainly composed of long-chain hydroxy fatty acids. These are excellent building blocks to produce new hydrophobic biopolymers. In this work, the monomers of cutin were extracted and isolated from tomato pomace and utilized to produce cutin-based films. Several strategies for the depolymerization and isolation of monomeric cutin were explored. Strategies differed in the state of the raw material at the beginning of the extraction process, the existence of a tomato peel dewaxing step, the type of solvent used, the type of alkaline hydrolysis, and the isolation method of cutin monomers. These strategies enabled the production of extracts enriched in fatty acids (16-hydroxyhexadecanoic, hexadecanedioic, stearic, and linoleic, among others). Cutin and chitosan-based films were successfully cast from cutin extracts and commercial chitosan. Films were characterized regarding their thickness (0.103 ± 0.004 mm and 0.106 ± 0.005 mm), color, surface morphology, water contact angle (93.37 ± 0.31° and 95.15 ± 0.53°), and water vapor permeability ((3.84 ± 0.39) × 10^−11^ mol·m/m^2^·s·Pa and (4.91 ± 1.33) × 10^−11^ mol·m/m^2^·s·Pa). Cutin and chitosan-based films showed great potential to be used in food packaging and provide an application for tomato processing waste.

## 1. Introduction

According to the Food and Agriculture Organization (FAO) of the United Nations, about one-third of all food resources produced for human consumption are lost [1]. This large fraction is due to the fact that food production is a wasteful process, with significant losses along its value chain, from crop residues through processing to sale and consumption [2].

Particularly, the fruit and vegetable processing industries generate a huge amount of itwaste every year [3]. These residues are extremely diverse due to the use of a wide variety of fruits and vegetables, representing excellent resources for a diversity of compounds, such as proteins, peptides, polysaccharides, dietary fiber, polyphenols, antioxidants, and natural pigments [3,4].

The tomato processing industry is a good example of this [3,4,5]. Tomato (*Solanum lycopersicum*) is one of the most produced and extensively consumed crops, after potatoes, with a worldwide rising production of 180 million tonnes in 2018, 183 million tonnes in 2019, and 186 million tonnes in 2020 [6,7]. Several applications have been exploited for the residue that arises from the tomato processing activity, designated as tomato pomace [3,4,5]. Among them are the use of this by-product as a supplement for animal feed [8,9,10], due to its high protein content, and the extraction of lycopene, an antioxidant, for incorporation into food, pharmaceuticals, and cosmetics [11,12,13]. More recently, the possibility of using this by-product for the extraction of a biopolymer, cutin, for the production of renewable materials has been explored [5,14,15].

Cutin represents 40 to 85% of the tomato cuticle’s dry weight [16]. This polymer is made up of long-chain (mainly C16 and C18) hydroxy fatty acids linked together by ester bonds [17]. Many of these monomers are polyhydroxylated, containing a terminal hydroxyl group but also medium-chain hydroxyl groups [14,18]. The presence of monomers with epoxy and oxo mid-chain groups is also reported [14,16,17,18]. The C16 dihydroxy fatty acids typically have the second hydroxyl group at C-10, C-9, C-8, and/or C-7, and the C18 dihydroxy fatty acids at C-9 and C-10 [19]. Cross-linking via these hydroxyl groups allows the formation of a rigid three-dimensional matrix characteristic of cutin [17]. Although in smaller amounts, the presence of aromatic groups, dicarboxylic acids, and glycerol are also reported [14,18].

Cutin’s hydrophobic, thermal, and mechanical properties have prompted consideration of the applicability of this material for the production of bioplastics as an alternative to the use of conventional plastics [5].

Production of cutin bioplastics requires the isolation of the cuticle from the raw materials (such as plant leaves, fruit peels, etc.). Cutin extraction is commonly achieved by its depolymerization, i.e., the breakdown into its constituent monomers, rather than extracting it as a whole. From the literature reported on this subject, it is noticeable that the cutin depolymerization procedure has been evolving from more complex, expensive, time-consuming, and non-green methods to simpler, more affordable, faster, and more sustainable methods [14]. Currently, simpler methodologies include direct hydrolysis of the material. Cigognini et al., 2015 [20], produced monomeric cutin extracts by direct hydrolysis of tomato peels with an alkaline sodium hydroxide solution. Marc et al., 2021 [21] also produced extracts enriched in cutin monomers from tomato peels by alkaline hydrolysis; however, prior to cutin depolymerization, peels were dewaxed with a mixture of acetone and ethanol.

The polysaccharide pectin was reported to be formulated with cutin to produce films by the casting method to mimic tomato peel [22]. Alternatively, the well-known polysaccharide chitosan is a promising candidate to produce films with cutin. Chitosan has been extensively studied in recent decades. Recently, one of the areas of focus had been the production of films and coatings for the preservation of fresh and processed foods, due to their excellent properties of antimicrobial activity, nontoxicity, biocompatibility, and biodegradability [23,24,25]. It is a derivative of chitin after deacetylation that can be extracted from shrimp shells and other crustaceans. Structurally, it is a linear polysaccharide composed of randomly distributed β-(1→4)-linked d-glucosamine and N-acetyl-d-glucosamine [23,24,25,26].

Pure chitosan films have already been tested in the preservation of various foods, including bananas, carrots, tomatoes, fish, and blueberries, among others. Delay of qualitative and nutraceutical alterations, prevention of the growth of microorganisms, maintenance of antioxidant activity, and extended shelf life were some of the positive effects observed on food when using chitosan-based films [23,24]. 

This macromolecule not only enables the production of pure films, but it is also a suitable compound to combine with other biopolymers to produce chitosan/biopolymer films. Some of these biopolymers include polysaccharides (e.g., starch, cellulose, pectin, etc.), proteins (e.g., caseinate, collagen, quinoa protein, etc.), and extracts (e.g., beeswax, citrus extract, olive oil, Silybum marianum L. extract, etc.) [23,27,28,29,30].

Considering the overwhelming amount of waste that results from tomato processing every year, the work developed in this study aimed to valorize this raw material through the production and characterization of cutin monomer-enriched extracts. After obtaining monomeric cutin extracts, their applicability in the production of hydrophobic films was studied. Films with cutin were developed together with a polysaccharide, chitosan, in a unique combination of two biopolymers in films not yet reported in the literature. Specifically, the polysaccharide chitosan was selected due to its excellent biocompatibility, biodegradability, film-forming, and antimicrobial properties commonly reported in the literature [23,24,25,30]. Despite all these favorable characteristics for its application in food packaging, chitosan-based films typically have a hydrophilic character, which translates into a decrease in their barrier properties when they are in the presence of water and in humid environments [31,32]. It is expected that the addition of hydrophobic materials such as fatty acids from tomato cutin may improve the moisture barrier properties, thus providing an advantage for these films. They were then characterized in terms of their optical properties, surface morphology, surface hydrophobicity, and barrier properties (against water vapor), envisaging their application in food packaging.

## 2. Materials and Methods

### 2.1. Materials

The raw material of the work was tomato pomace (Sumol, Compal, S.A., Almeirim, Portugal).

For the dewaxing of the tomato peels, ethanol (99.9%, Carlo Erba Reagents, Val de Reuil, France) and acetone (≥99.5%, Merck, Darmstadt, Germany) or n-heptane (99.0%, Carlo Erba Reagents, Val de Reuil, France) were used. For cutin depolymerization, NaOH pellets (98–100.5%, Sigma-Aldrich, St. Louis, MI, USA) or KOH pellets (99.99%, Sigma-Aldrich, St. Louis, MI, USA) were used and for the isolation and purification of the cutin monomers, HCl (≥37%, Honeywell, Wien, Austria) was used. For the monomeric cutin extracts’ characterization, methanol, sulfuric acid, and chloroform (HPLC-grade, Sigma-Aldrich, St. Louis, MO, USA) were used.

For the films’ production, chitosan (Golden-Shell Biochemical Co., Ltd., Zhejiang, China), glacial acetic acid (99.8%, Carlo Erba Reagents, Val de Reuil, France), glycerol (≥99.0%, Sigma-Aldrich, Darmstadt, Germany), ethanol (99.9%, Carlo Erba Reagents, Val de Reuil, France), and Tween^®^ 20 (≥40.0%, Sigma-Aldrich, St. Quentin Fallavier, France) were used. 

The water used in this work was purified using a Diwer Technologies purification unit.

For the isolation and purification of cutin monomers, as an alternative method to precipitation, filtration with two polyethersulfone PES membranes, Nadir^®^ NP010 P and Nadir^®^ NP030 P (Mann + Hummel Water & Fluid Solutions, Goleta, CA, USA) (Table 1).

### 2.2. Determination of the Moisture Content

To determine the moisture content of the tomato pomace, 3 samples of approximately 10 g of pomace were weighed and placed for a total of 120 h (5 days) at 60 °C in an oven (Venticell^®^ 111 Eco line, MMM Group, Planegg, Germany). Samples were taken out from time to time, and after the temperature dropped to room temperature, their weight was registered. When a stabilization of the weight was achieved, i.e., no significant changes in the weight values (inferior to 0.01 g), the moisture content (wet basis)—MC_wb_—of the samples was determined using Equation (1).

(1) $$MC_{wb}=\frac{m_{humid}-m_{dried}}{m_{humid}}\times 100$$

where *m_humid_* refers to the mass of the humid tomato pomace and *m_dried_* refers to the mass of the dried material.

### 2.3. Tomato Pomace Pre-Treatment

Tomato peels were separated from the remaining components of the tomato pomace (pulp, seeds, and fibers) by decantation in a water tank. The floating peels were recovered with a fine metallic mesh to drain off the excess water. 

The tomato peels were dried in a hot air oven (Venticell^®^ 111 Eco line, MMM Group, Planegg, Germany) for 48 h at 60 °C. Next, the dried tomato peels were milled into fine flakes using an analytical grinder (A10 basic, Ika, Staufen, Germany) to increase the transfer area between the extracting solvent and the solid material.

### 2.4. Tomato Peel Monomeric Cutin Extraction and Isolation

**Figure 1:**
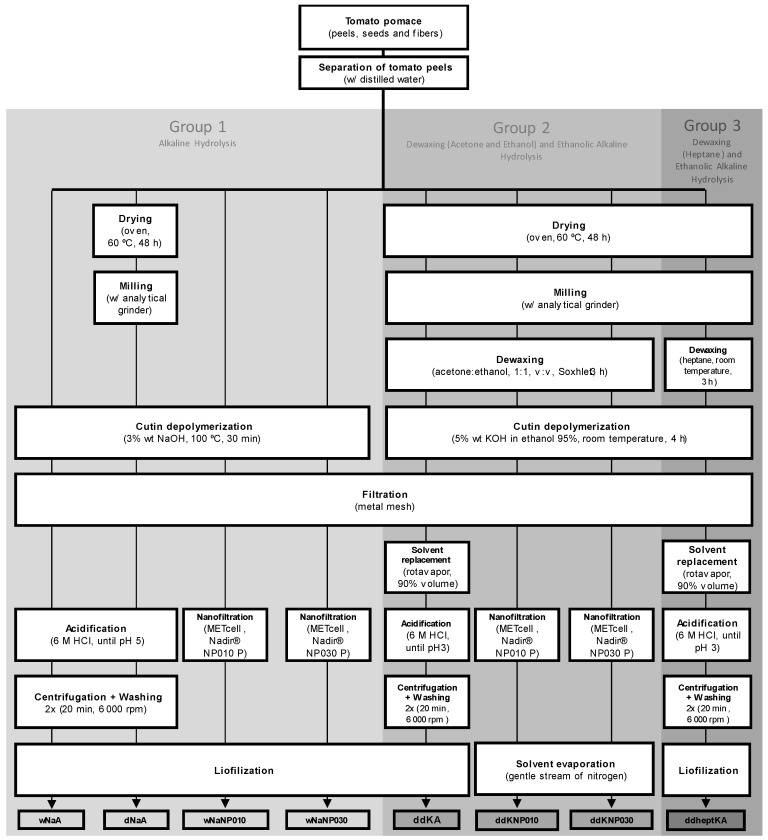
Flow diagram summarizing all the methods tested in this study to depolymerize and extract cutin. **Group 1 Alkaline Hydrolysis**: **wNaA**, wet tomato peels, depolymerization with NaOH, acidification; **dNaA**, dry tomato peels, depolymerization with NaOH, acidification; **wNaNP010**, wet tomato peels, depolymerization with NaOH, processed with NP010; **wNaNP030**, wet tomato peels, depolymerization with NaOH, processed with NP030. **Group 2 Dewaxing (Acetone and Ethanol) and Ethanolic Alkaline Hydrolysis**: **ddKA**, dry tomato peels, dewaxing with acetone and ethanol, depolymerization with KOH, acidification; **ddKNP010**, dry tomato peels, dewaxing with acetone and ethanol, depolymerization with KOH, processed with NP010; **ddKNP030**, dry tomato peels, dewaxing with acetone and ethanol, depolymerization with KOH, processed with NP030. **Group 3 Dewaxing (Heptane) and Ethanolic Alkaline Hydrolysis**: **ddheptKA**, dry tomato peels, dewaxing with heptane, depolymerization with KOH, acidification. Obs: For the sake of simplicity, not all processing steps are described in the legend, particularly: 1—after drying, there is a milling process; 2—after depolymerization, there is a filtration process; 3—after the acidification step, there is centrifugation and washing; 4—the final step is lyophilization, except during the processing sequence of Group 2, where membrane processing is the final step.

Several methods were tested to depolymerize and consequently isolate cutin from the tomato peels. All these methods are summarized in Figure 1.

#### 2.4.1. Group 1—Alkaline Hydrolysis

Cutin extraction was conducted according to the method proposed by Cigognini et al., 2015 [20], with some modifications. The tomato peels (*wet* or dried and milled, Figure 1) were mixed with a 3 wt.% NaOH solution (NaOH solution/tomato peels ≈ 4/1, *w*/*w*). The mixture was then heated at 100 °C for 30 min, under continuous stirring (300 rpm). To remove spent skins, the mixture was filtered through a fine metallic mesh, and the liquid fraction was collected. Cutin monomers were isolated and purified using one of two different methods-precipitation with an acidic solution or membrane processing (Figure 1). Precipitation of cutin monomers was promoted by adjusting pH to 5 (SensION™+ pH3, Hach, Düsseldorf, Germany) with a 6 M HCl solution. The extract enriched in cutin monomers was then recovered through centrifugation (Frontier™ Centrifuge FC5706, OHAUS, Zürich, Switzerland) of the suspension for 20 min at 6000 rpm, washed twice with Milli-Q water, and centrifuged likewise. Alternatively to precipitation, membrane filtration was performed in a MET^®^ Cell Dead-End filtration system (Evonik Membrane Extraction Technology Ltd., London, UK), under constant pressure (10 bar), using flat sheet membranes (membrane area of 51.4 cm^2^). During filtration, an electronic balance (Kern 572, KERN, Balingen, Germany) was used to measure the permeate mass. For each filtration, a total of 100 g of liquid extract enriched in cutin monomers was added to the system. The filtration process was conducted at room temperature under continuous stirring (400 rpm). The extracts were processed until a final Volume Reduction Factor VRF (the ratio between the initial feed volume and the retentate volume) of three was reached. Samples resulting from both methods—acidification and membrane processing—were freeze-dried (48 h treatment cycle) and then stored in a cold, dry, and dark environment until further use and/or analyses.

#### 2.4.2. Group 2—Dewaxing (Acetone and Ethanol) and Ethanolic Alkaline Hydrolysis

Cutin extraction was conducted according to the method proposed by Marc et al., 2021 [21], with some modifications. Dried and milled tomato peels were dewaxed under the reflux of acetone:ethanol 1:1 (*v*/*v*) in a Soxhlet extractor. The mass ratio of the extracting medium to the dried and milled tomato peels was 23:1, and the time of extraction was 3 h. Dewaxed tomato peels were dried overnight in a fume hood. For cutin depolymerization, the dewaxed tomato peels were mixed with 5 wt.% KOH in ethanol 95% (KOH in ethanol solution/tomato peels ≈ 17/1, *w*/*w*). The depolymerization was performed at room temperature, with constant stirring at 300 rpm for 4 h. To remove spent skins, the mixture was filtered through a fine metallic mesh, and the liquid fraction was collected. For three methods, one from Group 2 and two from Group 3 (see Figure 1), about 90% of the filtrate’s volume was evaporated under vacuum in a rotary evaporator (R-210, Buchi^®^, Flawil, Switzerland) at 35 °C and under reduced pressure (33 mbar). The volume that evaporated was replaced by water. As in Group 1, isolation and purification of cutin monomers were achieved with precipitation with an acidic solution or membrane processing. Precipitation of cutin was promoted by adjusting pH at 3.5 (SensION™+ pH3, Hach, Düsseldorf, Germany) with a 6 M HCl solution. Cutin was then recovered through centrifugation of the suspension for 20 min at 6000 rpm, washed twice with Milli-Q water, and centrifuged likewise. The membrane processing protocol was the same as for the methods in Group 1. Samples resulting from both methods—acidification and membrane processing—were freeze-dried (48 h treatment cycle) and then stored in a cold, dry, and dark environment until further use and/or analyses.

#### 2.4.3. Group 3—Dewaxing (Heptane) and Ethanolic Alkaline Hydrolysis

The cutin depolymerization methods of Group 3 are very similar to those of Group 2, as can be seen in Figure 1, except for the dewaxing step. In Group 3, the dried and milled tomato peels were dewaxed with heptane. For one of the methods, the dewaxing was performed at room temperature with constant stirring at 300 rpm for 3 h. For the other, dried and milled tomato peels were dewaxed under reflux in a Soxhlet extractor. The mass ratio of the extracting medium to the dried and milled tomato peels was 23:1, and the time of extraction was 3 h. Dewaxed tomato peels were dried overnight in a fume hood. Depolymerization was performed exactly as in Group 2 methods, as was the isolation and purification of cutin monomers through precipitation with an acidic solution. No membrane processing was performed in this group of methods. The resulting samples were freeze-dried (48 h treatment cycle) and then stored in a cold, dry, and dark environment until further use and/or analyses.

### 2.5. Characterization of the Monomeric Cutin Extracts (GC-FID)

The fatty acid content of the cutin monomer-enriched extracts was determined by GC-FID analysis. A first digestion step was performed following the method described by Lanham et al., 2013 [33], with some modifications. Briefly, 2–4 mg of each cutin lyophilized extract was incubated for methanolysis at 100 °C for 3.5 h with 1 mL of acidic methanol (20% sulfuric acid) and 1 mL of chloroform that contained nonadecanoic acid (1 g/L) as an internal standard (≥98%, Sigma-Aldrich, Darmstadt, Germany). After cooling, to separate the organic from the inorganic phase, 1 mL of Milli-Q water was added, and the mixture was vortexed for 1 min. The lower phases (organic phases) were extracted into 2 mL vials with molecular sieves to remove traces of water. After the digestion step, the organic phase (methylated monomers dissolved in chloroform) of each sample was extracted and injected into a gas chromatograph coupled to a Flame Ionization Detector (GC-FID 6890, Agilent Technologies, Inc., Santa Clara, CA, USA, and GC Autosampler HT3100A, HTA, Brescia, Italy). An OPTIMA 240 column (60 m, 0.25 mm ID, and 0.25 µm film) (Macherey-Nagel, Duren, Germany) was used at a flow rate of 1.5 mL/min. The mobile phase used was 33% cyanopropylmethyl–67% dimethy polysiloxane. The oven temperature program was as follows: 80 °C; then 20 °C/min until 120 °C; and finally, 3 °C/min until 260 °C. The detector temperature was set at 280 °C. Palmitic acid (≥99%), stearic acid (≥98.5%), oleic acid (≥99%), linoleic acid (≥99%), 16-hydroxyhexadecanoic acid (98%), hexadecanedioic acid (98%), and octadecanedioic acid (98%) concentrations were determined using commercial (Sigma-Aldrich, Darmstadt, Germany) external standards (concentration range of 0.03125–1 g/L) and corrected using a nonadecanoic acid as internal standard.

### 2.6. Determination of the Extraction Yields

The extraction yield was calculated for each of the monomeric cutin extracts using Equation (2).

(2) $$\mathrm{Yield}\;\left(\%\;w/w\right)=\frac{m_{freeze\text{-}dried\;extract}}{m_{dried\;tomato\;peels}}\times 100$$

In this equation, *m_freeze-dried extract_* refers to the mass of the freeze-dried monomeric cutin extract, and *m_dried tomato peels_* refers to the mass of the dried tomato peels from which the cutin has been extracted.

### 2.7. Preparation of Chitosan Solutions and Cutin Suspensions

The chitosan film-forming solution was prepared as described by Ferreira et al., 2016 [34], with certain modifications. Chitosan was dissolved in a glacial acetic acid solution (1% *w*/*w*) at a concentration of 1.5% *w*/*w* and left stirring overnight at room temperature. Glycerol (25% w_glycerol_/w_chitosan_) was added to the solution, followed by another 20 min of stirring for complete homogenization.

The monomeric cutin suspensions were prepared as previously described by Tedeschi et al., 2018 [15], with a few modifications. The two monomeric cutin extracts, with the highest mass percentage of total fatty acid per mass of freeze-dried extract, were added to a mixture of Milli-Q water and ethanol (1:1, *v*/*v*), at a concentration of 0.5% *w*/*w*. Tween^®^ 20 (25% w_Tween_/w_cutin_) was added, and the mixtures were dispersed by successive ultrasound cycles using a 6 mm diameter tapered microtip attached to a VCX750 ultrasonic processor (Sonics and Materials, Inc., Newtown, CT, USA).

#### 2.7.1. Preparation of the Chitosan Films (Control)

For the chitosan film (control), 10 g of the chitosan solution was cast onto a 50 mm diameter PFA (perfluoroalkoxy) evaporating dish (Bohlender, Grünsfeld, Germany) and dried overnight at 40 °C in an oven (Venticell^®^ 111 Eco line, MMM Group, Planegg, Germany). After being peeled from the dish, the film was stored and sealed in a glass petri dish and left in a desiccator at room temperature.

#### 2.7.2. Preparation of Cutin and Chitosan Blend Films

For the cutin and chitosan blend films, the monomeric cutin suspension was added to the chitosan solution (monomeric cutin suspension/chitosan solution ≈ 1/2, *w*/*w*), and the mixture was stirred for 20 min at room temperature until total homogenization. A mass of 15 g of the cutin and chitosan solution was cast onto the PFA evaporating dish (Bohlender, Grünsfeld, Germany). These were then left to dry overnight in a 40 °C oven (Venticell^®^ 111 Eco line, MMM Group, Planegg, Germany). After being peeled from the PFA evaporating dishes, the films were stored and sealed in glass Petri dishes and left in a desiccator at room temperature.

All films produced are listed in Table 2, along with the strategy used, composition of the chitosan solution and the cutin monomer suspension, and ultrasonic dispersion program.

### 2.8. Film Characterization

Before any of the following characterization procedures, the films were previously equilibrated at a relative humidity of 50.5% RH.

#### 2.8.1. Scanning Electron Microscopy (SEM)

The film’s surface morphology was analyzed by scanning electron microscopy (SEM). Samples were placed on aluminum stubs using double-sided carbon tape and were sputter-coated with a thin Au/Pd film with a Quorum Technologies coater (Q150T ES, E Hong Instruments Co., Ltd., Taipei, Taiwan). They were then analyzed with a Thermo Fischer Scientific (Waltham, MA, USA) desktop scanning electron microscope (Phenom ProX G6) equipped with an energy dispersive spectroscopy (EDS) light element.

#### 2.8.2. Thickness

The thickness of the films was measured with a digital micrometer (Filetta, Schut Geometrical Metrology, Groningen, The Netherlands). Measurements were made in three different places on the films.

#### 2.8.3. Color Measurements

To assess the films in terms of color, the CIELAB (or CIE L* a* b*) color system was used [35]. In this system, the parameter L* measures lightness, and its values range from 0 (black) to 100 (white). Parameters a* and b* measure the green/red and the blue/yellow color components, respectively. Negative values of a* (−a*) correspond to green, and positive values (+a*) correspond to red. The same happens for the coordinate b*; negative values of b* (−b*) correspond to blue, and positive values (+b*) correspond to yellow.

A digital colorimeter (Chroma Meter CR-400, Konica Minolta, Tokyo, Japan) was used to obtain the values of these three parameters (L*, a*, and b*) for each of the films. Five measurements of different areas of the films were performed after calibration of the colorimeter against a white calibration plate, where L* = 94.52, a* = −0.58, and b* = 3.79.

By obtaining these values, two other parameters were calculated—the hue (*h°*), which refers to the absorbance or reflection of specific wavelengths of light, and the chroma (C*), which indicates the saturation of color.

The *h°* was calculated using Equations (3), (4), or (5):

(3) $$h{}^{\circ}=\arctan\left(\frac{b^{*}}{a^{*}}\right)\times \frac{180}{\pi },\;\mathrm{for}\;a^{*}\;>\;0\;\mathrm{and}\;b^{*}\;>\;0$$

(4) $$h{}^{\circ}=\left(\arctan\left(\frac{b^{*}}{a^{*}}\right)\times \frac{180}{\pi }\right)+180,\;\mathrm{for}\;a^{*}\;<\;0$$

(5) $$h{}^{\circ}=\left(\arctan\left(\frac{b^{*}}{a^{*}}\right)\times \frac{180}{\pi }\right)+360,\;\mathrm{for}\;a^{*}\;>\;0\;\mathrm{and}\;b^{*}\;<\;0$$

The C* was calculated using Equation (6):

(6) $$C^{*}={\left({\left(a^{*}\right)}^{2}+{\left(b^{*}\right)}^{2}\right)}^{\frac{1}{2}}$$

The color difference ($\Delta E_{ab}^{*}$) was calculated (Equation (7)) in order to compare the color between each of the cutin and chitosan film samples and the control film sample (chitosan film).

(7) $$\Delta E_{ab}^{*}={\left({\left(\Delta L^{*}\right)}^{2}+{\left(\Delta a^{*}\right)}^{2}+{\left(\Delta b^{*}\right)}^{2}\right)}^{\frac{1}{2}}$$

If $\Delta E_{ab}^{*}$ >1 the color difference between the samples should be observable by the human eye [35].

#### 2.8.4. Water Contact Angle (WCA)

Water drop contact angles with the film surfaces were measured in order to access the film surface hydrophobicity. Measurements were performed with the Drop Shape Analyser—DSA25 (Krüss, Hamburg, Germany), using the Sessile Drop method. A 3 µL drop of distilled water was dropped onto the upper surface of previously cut squares of the film (1 cm × 1 cm). All measurements were performed at room temperature. Image analysis software Advance (Krüss) was used to calculate the contact angles of the drops, and the resulting values were given by the average on both sides of the drops. For each film type, three replicates were collected.

#### 2.8.5. Water Vapor Permeability (WVP)

The water vapor permeability (WVP) of the films was determined gravimetrically following the method used by Ferreira et al., 2016 [34], with slight modifications. Triplicates of flat circular film samples were sealed over cylindrical glass permeation cells with aluminum tape. The cylindrical glass cells (inner diameter = 40 mm) were previously filled with 9 mL of a saturated NaCl solution (a_w_ = 0.755).

After this, each of the permeation sets (one permeation set = cylindrical glass cell + NaCl solution + film sample + aluminum tape) was quickly weighed. Then all the sets were put in a desiccator containing a saturated MgCl_2_ solution (a_w_ = 0.328) and equipped with a fan to promote air circulation. The permeation sets were weighed at regular intervals for eight hours. During this time, the temperature and relative humidity inside the desiccator were measured with a thermohygrometer (Humicap^®^ HM40, Vaisala, Helsinki, Finland).

The WVP was calculated using Equation (8):

(8) $$\mathrm{WVP}=\frac{N_{w}\times \delta }{\Delta P_{w.eff}}$$

In this Equation, *N_w_* (mol/m^2^·s) is the water vapor flux, *δ* (m) is the film thickness, and ∆*P_w.eff_* (Pa) is the effective driving force, which was estimated according to the method used by Alves et al., 2010 [36].

## 3. Results

### 3.1. Moisture Content of Tomato Pomace and of Dry Matter Content of Its Fractions

The tomato pomace used in this study showed a high moisture content of 84.07 ± 0.42% (wet basis-w.b.). Similar or even higher moisture values were observed in other studies. Bhat and Ahsan, 2018 [37] reported a moisture content value of tomato pomace of 87.63 ± 0.12% (*w*/*w*), and Lavelli and Torresani, 2011 [11] obtained a moisture content value of 90.0 ± 0.2% (*w*/*w*).

The dry matter content of each fraction of tomato pomace, peels, seeds, and fibers was calculated (Table 3). Peels were the main fraction, followed by seeds and fibers.

In this study, the fraction with the highest content in dried matter was the peel fraction, which is in line with the results reported [12,38,39].

### 3.2. Tomato Monomeric Cutin Extracts

The extraction yield value was calculated for each of the monomer-enriched cutin extracts produced (Table 4).

The cutin extraction methods investigated showed significant differences in terms of extraction yield.

By comparing the extraction yield values of samples obtained with NaOH hydrolysis with different moisture contenst, wet or dried (wNaA and dNaA), one can infer that the sample in which tomato peels were dried and milled prior to cutin depolymerization (sample dNaA) had the highest yield. This may suggest that the efficiency of the depolymerization reaction is influenced by the moisture content of the material soon to be hydrolyzed and the surface area between the solvent and the solid material.

The effect of cutin isolation methods, particularly membrane processing and acidification for cutin precipitation, was also studied. Within Group 1, the samples with the same initial moisture content (wet tomato peels), the same depolymerization method (with NaOH), and processed by membranes, the samples wNaNP010 and wNaNP030, exhibited a higher cutin extraction yield when compared to the sample processed by acidification (wNaA). Within Group 2, the samples with the same initial moisture content (dry tomato peels), the same dewaxing method (acetone and ethanol), the same depolymerization method (with KOH), and processed by membranes, the samples ddKNP010 and ddKNP030, exhibited a higher cutin extraction yield when compared to the sample processed by acidification (sample ddKA).

The sample dewaxed with heptane at room temperature (ddheptKA from Group 3) had a slightly higher cutin yield extraction value than that obtained for the sample from KOH hydrolysis (ddKA from Group 2).

Cigognini et al., 2015 [20] reported a 15% (*w*/*w*) yield value using NaOH hydrolysis, the method used in this work to produce sample wNaA, for which a lower value of 6.74% (*w*/*w*) was obtained. This difference may be due to either the time or the temperature values chosen for the depolymerization reaction, or more likely both. In this work, the reaction was run for 30 min at 100 °C, while the authors let the reaction run for 2 h without specifying the temperature used, giving only a range.

The extraction yield value of the sample using KOH hydrolysis (ddKA) was 21.11% (*w*/*w*), significantly lower than the 60% (*w*/*w*) obtained by Marc et al., 2021 [21]. However, this difference can be directly related to the reaction time of the depolymerization step. While Marc et al., 2021 [21] left the reaction running for 24 h, in this study only the effect of 4 h of depolymerization was studied.

Despite this, ethanolic alkaline hydrolysis at room temperature (ddKA) led to a higher extraction yield of monomeric cutin extract (21.11% *w*/*w*) than that for aqueous alkaline hydrolysis at 100 °C (samples wNaA and dNaA), 6.74 and 16.46% (*w*/*w*), respectively, which may be a very interesting finding in terms of process sustainability.

### 3.3. Fatty Acid Composition

The results of the fatty acid composition of the monomeric cutin extracts investigated by GC-FID, are presented in Table 5 (results given as % (w_fatty acid_/w_total selected fatty acids_)) and Table 6 (results given as % (w_fatty acid_/w_freeze-dried extract_)).

Considering the results presented in Table 5, the major fatty acid identified was 16-hydroxyhexadecanoic acid, with the highest percentage (w_fatty acid_/w_total selected fatty acids_) in seven out of the eight samples analyzed, ranging from 62.05% in the sample obtained from KOH hydrolysis and acidification (ddKA) to 8.07% in the sample obtained from NaOH hydrolysis of dried tomato peels and acidification (dNaA). Followed by hexadecanedioic acid, the second major fatty acid in six out of the eight samples, with a range of values from 17.54% (sample wNaA) to 10.51% (sample ddKA). Stearic, linoleic, palmitic, and octadecanedioic acids have also been identified, although in smaller amounts than the previous fatty acids. The component with the lowest percentage (w_fatty acid_/w_total selected fatty acids_) in seven out of the eight samples analyzed was oleic acid, ranging from 10.71% in sample dNaA to 2.82% in sample ddKA.

On a more thorough level, when analyzing samples that are within the same group, there are also some observations that can be drawn. When comparing samples wNaA and dNaA (Group 1), whose difference in the extraction method is based on the use of humid tomato peels (sample wNaA) or dried and milled tomato peels (sample dNaA), it is evident that there are differences in the fatty acid profiles of the two samples. Although the second major component, hexadecanedioic acid, was the same for both samples, representing 17.54% of the total fatty acids investigated in sample wNaA and 15.41% in sample dNaA, that was not the case for the most predominant compound. The major fatty acid identified in sample wNaA was 16-hydroxyhexadecanoic with a total percentage of 23.66%. In sample dNaA, on the other hand, it was palmitic acid, representing 31.04% of total fatty acids.

Therefore, it is possible that the drying and milling pre-treatment of tomato peels may influences the final fatty acid profile in the sense that the existence of this pre-treatment may contribute to more or less complete depolymerization of cutin.

In addition, within Group 1, for samples resulting from hydrolysis with NaOH and processed by membranes (wNaNP010 and wNaNP030), very similar fatty acid profiles were obtained. It should be noted that the only difference between the extraction methods that originated the two samples was the use of a membrane with a smaller pore size in the case of sample wNaNP030 (MWCO of 500–1000 Da), and a larger pore size in the case of sample wNaNP010 (MWCO of 1000–1500 Da). This may indicate that the membrane processing step was not efficient in isolating the cutin monomers. Combining the cutin extraction yield values (Table 4) with the fatty acid profiles further supports this hypothesis. Samples wNaNP010 and wNaNP030 have extraction yields of 38.83 and 28.48% (w_freeze-dried extract_/w_dried residue_), respectively, while sample wNaA has a yield value of 6.74%. However, samples wNaNP010 and wNaNP030 do not show a higher weight percentage of fatty acids per weight of freeze-dried extract (Table 6). Thus, this supports what was previously indicated in the previous section, suggesting that the membrane processing step was not more efficient when compared to the alternative method of cutin monomer isolation-precipitation with an acidic solution (sample wNaA).

It can also be observed with samples from Group 2. The samples dKNP010 and dKNP030 had extraction yield values that were four times higher than those of ddKA. However, these values do not translate into higher values of fatty acid concentration.

These results are in agreement with those reported in other studies [5,16,18], in the sense that the presence of many of the same compounds was detected in the extracts produced, namely 16-hydroxyhexadecanoic acid, linoleic acid, oleic acid, palmitic acid, etc.

The samples selected for the study of the cutin-based film production were the samples dNaA (Group 1) and ddKA (Group 2) because these samples had the highest mass percentage of total fatty acid per mass of freeze-dried extract.

### 3.4. Cutin and Chitosan Films

All films were produced by the casting method and are shown in Table 7.

Film sample (1) resulted from the casting of a solution of chitosan dissolved in acetic acid (1.5% *w*/*w*), which contained glycerol as a plasticizer.

Film samples (2) and (3) were produced by drying a blend of a 1.5% *w*/*w* chitosan solution with a suspension of cutin monomers—monomeric cutin extract ddKA or dNaA—in water and ethanol and containing the dispersing agent Tween^®^ 20.

After being removed from the oven, where the films dried at 40 °C overnight, all films were easily peeled from the PFA evaporating dishes.

Of the chitosan films produced (Table 7), film sample (1) had smooth, uniform, and homogeneous surfaces. These films were flexible, very easy to handle, and visually appeared to be transparent with a slightly yellowish coloration.

Of the cutin and chitosan films (Table 7), film samples (2) and (3) displayed a dark yellow/orange color as a result of adding the cutin extracts (Table 4) to the solution. These blended films (2) and (3) were malleable and intact. Even so, when comparing both, film (2) had the smoothest, most uniform, and most homogeneous surface detectable to the naked eye. It is very likely that these observable differences in the two film samples are due to the monomeric cutin extracts that each of these films has in their composition. Differences between the two cutin extraction methods that originated dNaA and ddKA extracts, such as the existence of a dewaxing step and the type of alkaline solution and temperature at which the depolymerization of the cutin occurred, most likely contributed to the formation of different monomeric cutin extracts and, consequently, resulted in films with different appearances.

#### 3.4.1. Film Morphology

The film’s surface morphology, analyzed by scanning electron microscopy (SEM), is shown in Table 8.

The images acquired by SEM corroborate what was observed with the naked eye (Section 3.4), namely that the chitosan control film (Table 8, images A1 and A2) exhibits a smooth, uniform surface with no visible cracks. This morphology is consistent with that usually obtained for other chitosan films [34]. In comparison to the chitosan control film (Table 8, images A1 and A2), the cutin and chitosan films (2) and (3) (Table 8, images B1, B2, C1, and C2) show a more irregular structure. This characteristic can possibly be attributed to some aggregates of cutin monomers that were not completely dissolved. This was to be expected in the case of film (3), since the cutin suspension was not completely homogeneous.

#### 3.4.2. Thickness

The thickness values of the films are listed in Table 9.

The chitosan film (control)—film sample (1)—presented the lowest thickness value, being the thinnest film produced in this work, with an average thickness of 0.092 ± 0.008 mm. Cutin and chitosan film samples (2) and (3) showed slightly higher thickness values, respectively, of 0.103 ± 0.004 and 0.106 ± 0.005 mm.

These results were as expected since the cutin and chitosan films were produced with the same mass of chitosan film-forming solution as the control film, i.e., 10 g of chitosan filmogenic solution. Therefore, the difference in thickness between these films and the control should be mostly due to the non-volatile matter of the monomeric cutin suspensions added. Additionally, it was also anticipated that the thickness value of film sample (2) would be very close to that of film sample (3) since the only difference in the composition of the two films is the depolymerized cutin extract used. For the production of film sample (2), the monomeric cutin extract used was ddKA, and for film sample (3), was dNaA.

#### 3.4.3. Color

The exact colors of the films were determined according to the CIELAB color system.

The measured (L*, a*, and b*) and the calculated (*h°* and C*) color parameters are summarized in Table 10.

For the chitosan control film sample (1), a very high lightness (L*) value was obtained (92.72). This value was very close to 100 (white) and, therefore, very far from 0 (black), indicating that the film had a very light shade. As for the a* and b* coordinates, this film presented a negative a* value (−1.99) and a positive b* value (10.50), resulting in a *h°* value of 100.72 and a C* value of 10.68. These values confirm what was observed with the naked eye, i.e., that the films had a yellowish tone with a low color saturation.

These color results for the chitosan-based films match those produced by Leceta et al., 2013 [25], especially the films produced with low molecular weight (L_Mw_) chitosan and 30% w_glycerol_/w_chitosan_.

The color parameters of the cutin and chitosan film samples (2) and (3) varied, as expected, from the chitosan control film. Both samples, (2) and (3), had lower L* values, which is consistent with the film’s darker colors observed visually. Unlike the control film, these two samples showed positive values of the a* coordinate, which indicates a color transition from green to red tones. This was completely expected given that orange/red extracts of depolymerized cutin were added to the chitosan film-forming solutions for the production of these films. On the other hand, the control film and the samples both revealed positive b* coordinates, but significantly higher values in the cutin and chitosan samples, indicating that these films have a more intense yellow color component. These differences in a* and b* coordinate values resulted in lower *h°* values for samples (2) and (3). Finally, higher chroma (C*) values were obtained for the cutin and chitosan film samples, mirroring the increase in color saturation that could be observed when comparing these to the control film.

**Figure 2:**
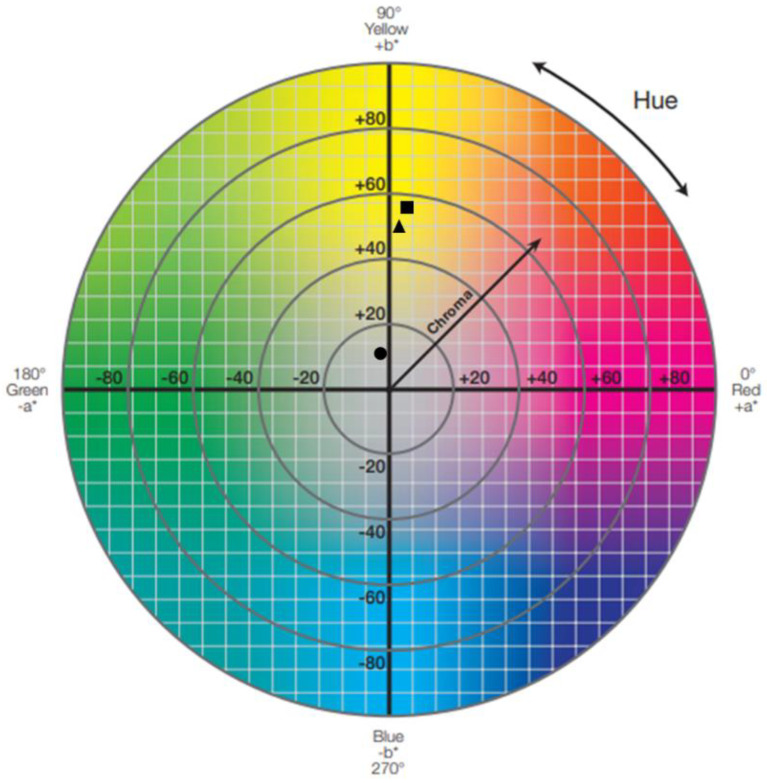
Graphic representation of the color of the three films, (1), (2), and (3), in the CIELAB hue circle (adapted from [40]). Legend: ●—film sample (1): 1.5% chitosan (control); ■—film sample (2): 1.5% chitosan + 0.5% cutin ddKA + Tween^®^ 20; ▲—film sample (3): 1.5% chitosan + 0.5% cutin dNaA + Tween^®^ 20.

By obtaining these color parameters, in particular the a* and b* chromaticity coordinates, it was possible to determine the approximate color position of these films in the CIELAB hue circle (Figure 2).

As expected, the position of the chitosan control film is in the second quadrant of the graph, a quadrant that ranges from yellow to green, while the two cutin and chitosan films are in the first quadrant, which ranges from red to yellow.

To thoroughly evaluate how the incorporation of the depolymerized cutin suspension changed the color of the cutin and chitosan films compared to the chitosan control film and to what extent this change is detectable to the human eye, the color difference (∆E*ab) between the films was calculated (Table 11).

The $\Delta E_{ab}^{*}$ values obtained were well above one, more precisely 50.07 for film sample (2) and 42.60 for film sample (3). This confirms a significant variation in the colors of the cutin and chitosan films compared to the chitosan film and that this can be clearly perceived by the human eye.

#### 3.4.4. Water Contact Angle

In order to assess the surface hydrophobicity of the produced films, the static contact angles between a water droplet and the upper surface of each of the films were measured (Table 12).

The measurements were performed on the upper surface of the films, so the data shown above has no influence on the flat PFA evaporating dish surface where the films were dried. The measurement of the contact angle with a solvent is a way to evaluate the affinity between that solvent and the surface of the material being tested.

Leceta et al., 2013 [25] reported a water contact angle result similar to that obtained for the control film of this study, reaching a value between 90 and 95° for low molecular weight (L_Mw_) chitosan and 30% w_glycerol_/w_chitosan_ films.

The results gathered in Table 12 suggest that all the samples revealed similar static water contact angle values for both the control and the cutin and chitosan films. Since high contact angles are characteristic of hydrophobic surfaces, these results suggest that the films produced have a hydrophobic character, even though the incorporation of cutin did not produce a significant increase in the water contact angle of the films, as one might expect.

#### 3.4.5. Water Vapor Permeability

The water vapor permeability (WVP) results for chitosan (control) and cutin and chitosan blend films for a driving force of 75.5–32.8% RH (relative humidity) are presented in Table 13.

The WVP parameter is a very important parameter, especially if these films are to be used for food packaging. However, it is also a very challenging parameter to compare, since the WVP is highly dependent on a number of factors, including film thickness, the driving force used in the assay, the concentration and type of plasticizer used, other additives added, the molecular weight of the polymer, etc. [34].

The chitosan film (control) presented a WVP value of (5.33 ± 1.66) × 10^−11^ (mol·m/m^2^sPa), which was higher than the 4.13 ± 0.13 × 10^−11^ (mol·m/m^2^sPa) value reported by Ferreira et al., 2016 [34] for a film with 1.5% (*w*/*w*) chitosan, 30% w_glycerol_/w_chitosan_ and 50% w_citric acid_/w_chitosan_ and a driving force of 76.9–22.5% RH.

The cutin and chitosan film samples (2) and (3) obtained the lowest values of WVP, of 3.84 ± 0.39 × 10^−11^ and 4.91 ± 1.33 × 10^−11^ (mol·m/m^2^sPa), respectively.

Tedeschi et al., 2018 [15] reported an improvement in the barrier properties, including WVP, of films composed of sodium alginate, fatty acids from tomato pomace, and beeswax. The authors observed a decrease in the WVP parameter with the proportion of tomato pomace monomers, attributing this result to the hydrophobic nature of the polyhydroxylated and unsaturated long-chain fatty acids of tomato pomace.

The results obtained for film samples (2) and (3) match those obtained by Tedeschi et al., 2018 [15], despite the fact that the WVP value obtained for film sample (3) is only slightly lower than that obtained for control film (1), which may be related to the lower uniformity and homogeneity of this film when compared to film (2) as previously observed (Section 3.4.1).

## 4. Conclusions

The massive increase in food processing waste and the urgent need to replace conventional plastics in packaging applications are major problems of the present time. This work contributed to the development of an alternative involving biodegradable materials based on cutin and to replace conventional plastics for packaging.

Tomato pomace, a waste product from the tomato processing industry, was characterized regarding its moisture content (84.07 ± 0.42% *w*/*w*) and its fractional composition (63.62 ± 1.85% peel, 34.59 ± 2.51% seed, and 1.80 ± 0.73% fiber, *w*/*w*).

Several methods for extracting and depolymerizing cutin, a biopolymer of the tomato cuticle, were explored. Methods included common processes, affordable chemicals, such as aqueous solutions of NaOH, KOH, and HCl, and membrane processing.

Monomeric extracts of cutin were successfully obtained and confirmed to be enriched in several fatty acids, including 16-hydroxyhexadecanoic acid, hexadecanedioic acid, stearic acid, and linoleic acid, among others.

Cutin and chitosan-based films were prepared from the extracts of recovered tomato cutin monomers and commercial chitosan by casting and drying at 40 °C. Cutin and chitosan blend films were malleable, homogeneous, hydrophobic (water contact angle values higher than 90°), and had high water vapor permeability.

The hydrophobic films produced in the course of this study undoubtedly show great potential, even though some improvements are needed before starting to replace food packaging made from petroleum-based polymers. In addition, they may provide an application for tomato processing waste, making the process of tomato production and processing more profitable and sustainable.

This study was mainly focused on the optimization of the cutin extraction process, ending with a preliminary characterization of cutin and chitosan-based films. In future work, a deeper study will be carried out to thoroughly characterize the films in terms of their mechanical properties, barrier properties to gases, and antimicrobial activity.

## Figures and Tables

**Table 1:** Characteristics of the membranes used in this study, according to the manufacturers’ data.

Membrane	Material	MWCO ^a^ (Da)	pH Range	Maximum Pressure (Bar)
Nadir^®^ NP010 P	PES ^b^ on PP ^c^	1000–1500	0.0–14.0	40
Nadir^®^ NP030 P	PES ^b^ on PP ^c^	500–1000	0.0–14.0	40

^a^ MWCO—Molecular Weight Cut-Off; ^b^ PES—Polyethersulfone; ^c^ PP—Polypropylene.

**Table 2:** Chitosan solution and cutin suspensions used to produce cutin and chitosan blend films.

Film Sample	Chitosan Solution Composition		Monomeric Cutin Suspension Composition		
	Chitosan (w_chitosan_/w_solution_)	Glycerol (w_glycerol_/w_chitosan_)	Cutin monomers (w_cutin monomers_/w_suspension_)	Tween^®^ 20 (w_Tween_/w_cutin_)	Ultrasonic Dispersion
(1)	1.5%	25%	-	-	-
(2)	1.5%	25%	0.5%	25%	3× (1 min, 40% amplitude)
(3)	1.5%	25%	0.5%	25%	3× (1 min, 40% amplitude)

**Table 3:** Peel, seed, and fiber ratio values determined for the batch of tomato pomace used in the present work. Results are given as % (w_dried component_/w_dried tomato pomace_).

Fraction	Dried Component, % (w_dried component_/w_dried tomato pomace_)
Peel	63.62 ± 1.85
Seed	34.59 ± 2.51
Fiber	1.80 ± 0.73

**Table 4:** Extraction yields of monomeric cutin from tomato peels. Results are given as % (w_freeze-dried extract_/w_dried tomato peels_). **Group 1 Alkaline Hydrolysis**: **wNaA**, wet tomato peels, depolymerization with NaOH, acidification; **dNaA**, dry tomato peels, depolymerization with NaOH, acidification; **wNaNP010** wet tomato peels, depolymerization with NaOH, processed with NP010; **wNaNP030**, wet tomato peels, depolymerization with NaOH, processed with NP030. **Group 2 Dewaxing (Acetone and Ethanol) and Ethanolic Alkaline Hydrolysis**: **ddKA**, dry tomato peels, dewaxing with acetone and ethanol, de-polymerization with KOH, acidification; **ddKNP010**, dry tomato peels, dewaxing with acetone and ethanol, depolymerization with KOH, processed with NP010; **ddKNP030**, dry tomato peels, dewaxing with acetone and ethanol, depolymerization with KOH, processed with NP030. **Group 3 Dewaxing (Heptane) and Ethanolic Alkaline Hydrolysis**: **ddheptdKA**, dry tomato peels, dewaxing with heptane, depolymerization with KOH, acidification.

	Group 1				Group 2			Group 3
Extraction Yield, % (*w*/*w*)	wNaA	dNaA	wNaNP010	wNaNP030	ddKA	ddKNP010	ddKNP030	dheptKA
	6.74	16.46	38.83	28.48	21.11	88.16	82.00	26.64

**Table 5:** GC-FID analysis of the constituents identified in cutin samples. Results are given as % (w_fatty acid_/w_total selected fatty acids_).

	Group 1				Group 2			Group 3
Fatty Acid	wNaA	dNaA	wNaNP010	wNaNP030	ddKA	ddKNP010	ddKNP030	dheptKA
**Palmitic acid**	14.47	31.04	12.26	12.27	6.33	11.52	11.85	10.74
**Stearic acid**	10.44	14.32	16.30	16.62	5.61	13.07	13.57	7.45
**Oleic acid**	6.31	10.71	7.87	7.84	2.82	6.91	7.07	4.61
**Linoleic acid**	16.29	11.97	12.94	12.41	7.22	12.71	12.54	12.68
**Hexadecanedioic acid**	17.54	15.41	15.53	14.81	10.51	14.61	15.08	13.38
**16-hydroxyhexadecanoic acid**	23.66	8.07	20.67	21.64	62.05	28.48	26.70	43.41
**Octadecanedioic acid**	11.30	8.49	14.43	14.41	5.46	12.70	13.19	7.73

**Table 6:** GC-FID analysis of the constituents identified in cutin samples. Results are given as % (w_fatty acid_/w_freeze-dried extract_).

	Group 1				Group 2			Group 3
Fatty acid	wNaA	dNaA	wNaNP010	wNaNP030	ddKA	ddKNP010	ddKNP030	ddheptKA
**Palmitic acid**	1.86	6.55	0.80	0.78	1.71	1.21	1.01	1.96
**Stearic acid**	1.34	3.02	1.06	1.05	1.52	1.37	1.15	1.36
**Oleic acid**	0.81	2.26	0.51	0.50	0.76	0.72	0.60	0.84
**Linoleic acid**	2.09	2.53	0.84	0.79	1.95	1.33	1.07	2.31
**Hexadecanedioic acid**	2.25	3.25	1.01	0.94	2.84	1.53	1.28	2.44
**16-hydroxyhexadecanoic acid**	3.03	1.70	1.35	1.37	16.76	2.99	2.27	7.90
**Octadecanedioic acid**	1.45	1.79	0.94	0.91	1.47	1.33	1.12	1.41
**Total selected fatty acids**	12.82	21.10	6.52	6.33	27.01	10.49	8.50	18.21

**Table 7:** Composition of film-forming solution and visual appearance of the films produced in this work.

Film Sample	Image
(1) 1.5% Chitosan (control)	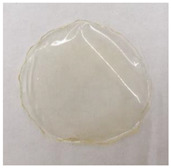
(2) 1.5% Chitosan + 0.5% Cutin ddKA (dry tomato peels; dewaxing with acetone and ethanol; depolymerization with KOH, acidification)	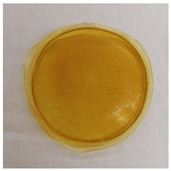
(3) 1.5% Chitosan + 0.5% Cutin dNaA (dry tomato peels, depolymerization with NaOH, acidification)	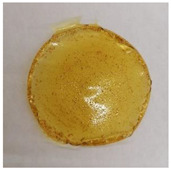

**Table 8:** SEM images of the surfaces of all the film samples.

Film sample	Magnification (Pol)	
	500×	1000/3000×
(1) 1.5% Chitosan (control)	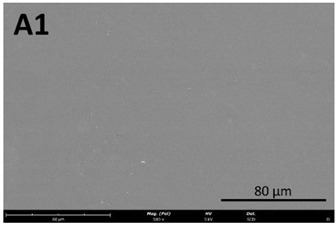	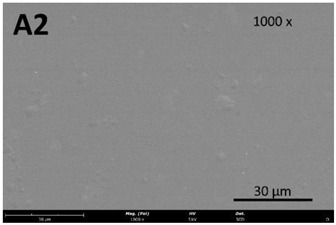
(2) 1.5% Chitosan + 0.5% Cutin ddKA	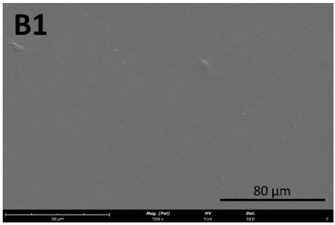	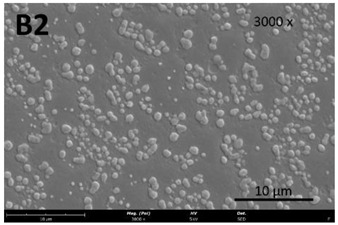
(3) 1.5% Chitosan + 0.5% Cutin dNaA	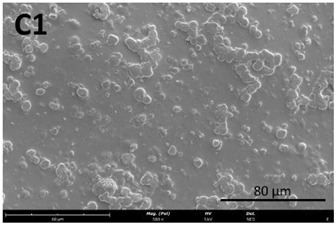	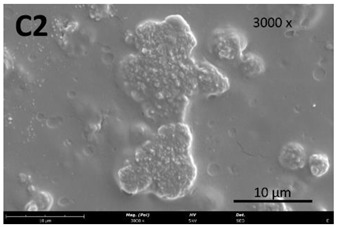

**Table 9:** Thickness (mm) of the selected film samples.

Film Sample	Thickness (mm)
(1) 1.5% Chitosan (control)	0.092 ± 0.008
(2) 1.5% Chitosan + 0.5% Cutin ddKA	0.103 ± 0.004
(3) 1.5% Chitosan + 0.5% Cutin dNaA	0.106 ± 0.005

**Table 10:** Measured color parameter values: lightness (L*), chromaticity coordinates (a* and b*), and calculated hue (*h*°) and chroma (C*) of the selected film samples.

Film Sample	L*	a*	b*	*h°*	C*
(1) 1.5% Chitosan (control)	92.72 ± 0.77	−1.99 ± 0.29	10.50 ± 1.47	100.72 ± 0.19	10.68 ± 1.49
(2) 1.5% Chitosan + 0.5% Cutin ddKA	72.18 ± 2.43	4.99 ± 2.13	55.63 ± 0.69	84.86 ± 2.25	55.89 ± 0.52
(3) 1.5% Chitosan + 0.5% Cutin dNaA	76.38 ± 0.95	2.52 ± 0.79	49.58 ± 1.42	87.10 ± 0.83	49.65 ± 1.45

**Table 11:** Color difference ($\Delta E_{ab}^{*}$) values between the chitosan film sample (1) and the two selected cutin and chitosan film samples (2) and (3).

Film Sample	$\mathrm{Color}\;\mathrm{Difference}\;(\Delta E_{ab}^{*})$
(1) 1.5% Chitosan (control) *&* (2) 1.5% Chitosan + 0.5% Cutin ddKA	50.07 ± 1.82
(1) 1.5% Chitosan (control) *&* (3) 1.5% Chitosan + 0.5% Cutin dNaA	42.60 ± 1.93

**Table 12:** Static water contact angle values (*θ*, °) of the film samples.

Film Sample	*θ* (°)
(1) 1.5% Chitosan (control)	93.35 ± 0.14
(2) 1.5% Chitosan + 0.5% Cutin ddKA	93.37 ± 0.31
(3) 1.5% Chitosan + 0.5% Cutin dNaA	95.15 ± 0.53

**Table 13:** Water Vapor Permeability values of the selected film samples.

Film Samples	WVP (×10^−11^ mol·m/m^2^sPa)
(1) 1.5% Chitosan (control)	5.33 ± 1.66
(2) 1.5% Chitosan + 0.5% Cutin ddKA	3.84 ± 0.39
(3) 1.5% Chitosan + 0.5% Cutin dNaA	4.91 ± 1.33

## Data Availability

Not applicable.
